# Characteristics and outcomes of patients with endometriosis and malignant or borderline ovarian tumors: real-world evidence from an ESGO centre of excellence

**DOI:** 10.1186/s12885-026-15980-w

**Published:** 2026-04-16

**Authors:** Maximilian Heinz Beck, Paul Kordowitzki, Eva Roser, Anna Trelinska-Finger, Emily Schoof, Lukas Chinczewski, Sylvia Mechsner, Jalid Sehouli

**Affiliations:** 1 Department of Gynecology with Center for Oncological Surgery,Campus Virchow Klinikum, Charité-Universitätsmedizin Berlin, Berlin, Germany; 2 Department of Basic and Preclinical Sciences, Nicolaus Copernicus University, Torun, Poland; 3 Clinical Cancer Registry of Charité, Charité Comprehensive Cancer Center, Berlin, Germany

**Keywords:** Endometriosis, Ovarian Cancer, Endometriosis associated Ovarian Cancer, Endometriosis correlated Ovarian Cancer, Endometriosis incidental Ovarian Cancer, Ovarian Tumor

## Abstract

**Background:**

Endometriosis is associated with an increased risk of type I ovarian cancer, yet the prognostic relevance of concurrent endometriosis at diagnosis remains unclear. This cohort study evaluated whether endometriosis independently influences survival outcomes in patients with ovarian cancer and investigated the clinicopathologic characteristics of endometriosis-associated ovarian tumors.

**Methods:**

This retrospective cohort study included patients treated for primary malignant or borderline ovarian tumors between January 2014 and July 2025 at a tertiary ESGO-accredited German academic center. Concurrent endometriosis was defined by histologic confirmation at primary surgery and classified as endometriosis-correlated ovarian tumors when histopathologic a transitional lesion from endometriosis to tumor was identified, or as endometriosis-incidental ovarian tumors when endometriosis was identified without transitional lesions. An age-matched reference cohort without endometriosis (1:5) was used for comparison. Survival analyses were restricted to patients with epithelial ovarian cancer and performed using Kaplan–Meier estimates and multivariable Cox regression, adjusting for age, histologic subtype, FIGO stage, endometriosis status, and surgical outcomes.

**Results:**

Among 2,164 eligible patients, 176 (8.1%) had histologically confirmed endometriosis, including 22 (1.0%) endometriosis-correlated ovarian tumors and 154 (7.1%) endometriosis-incidental ovarian tumors. Patients with endometriosis were significantly younger and more frequently diagnosed with early-stage disease and endometrioid or clear cell histology compared with patients without endometriosis. Endometriosis-correlated ovarian tumors showed a high prevalence of ovarian endometriosis, the absence of p53 aberrations, and no BRCA1/2 mutations. Median overall survival was not reached for patients with endometriosis-correlated ovarian tumors or endometriosis-incidental ovarian tumors. After multivariable adjustment, neither endometriosis-correlated ovarian tumors nor endometriosis-incidental ovarian tumors was independently associated with overall or disease-free survival. Advanced FIGO stage, older age, and incomplete cytoreduction were independently associated with worse survival.

**Conclusions:**

Endometriosis-associated ovarian tumors show favorable clinicopathologic features but concurrent endometriosis is not an independent prognostic factor for survival in patients with ovarian cancer. Prognosis appears to be driven primarily by tumor biology, stage, and surgical outcome rather than the presence of endometriosis itself.

**Supplementary Information:**

The online version contains supplementary material available at 10.1186/s12885-026-15980-w.

## Background

Endometriosis is a chronic, potentially disabling, estrogen-dependent inflammatory condition characterized by the presence of endometrial-like tissue outside the uterine cavity and affects approximately 5–10% of women of reproductive age [1, 2]. In recent decades, endometriosis has increasingly been discussed for its association with malignant ovarian neoplasms [3]. Large population-based studies have shown that women with ovarian endometriomas and/or deep infiltrating endometriosis may have an increased risk of ovarian cancer [4]. The relative risk of ovarian cancer among women with deep infiltrating endometriosis has been estimated to be 2- to 10-fold higher than in women without endometriosis, with the highest risk reported for patients with ovarian endometriosis [5–9]. This risk was primarily associated with type I ovarian cancer, such as endometrioid, clear cell or low-grade serous subtypes [4–6, 9–11]. Consistent with these findings, genomic liability to endometriosis has also been associated with risk for ovarian cancer [12].

Although benign, endometriosis shares several hallmarks of malignancy, including tissue invasion, metastasis, angiogenesis, and reduced apoptosis [13, 14]. These cancer-like characteristics have led to intensive research on molecular pathways that drive disease progression and potential malignant transformation in endometriosis. Atypical endometriosis has been identified as a premalignant precursor lesion and is characterized by dysplastic features such as cellular atypia, distinguishing it from typical endometriosis [15]. It has been reported in 12–35% of ovarian endometriomas and precedes 60–80% of endometriosis-associated ovarian cancers [16]. The stepwise progression - from benign ectopic endometrial epithelium to atypical changes and ultimately invasive carcinoma - is driven by complex molecular and microenvironmental alterations such as ARID1A mutation and early activation of the PI3K/AKT/mTOR pathway [17–19]. Estrogen overstimulation, tumor suppressor gene inactivation, and oxidative stress have been discussed as major drivers of this transformation [10].

Endometriosis-associated ovarian cancer was first described by Sampson in 1925 and further refined by Scott in 1953, who defined endometriosis-associated carcinoma by the presence of benign endometrial tissue contiguous with malignant epithelium and histological evidence of a direct transition [20]. In recent years a redefinition of endometriosis-associated ovarian cancer has been introduced by Mezzapesa and colleagues, distinguishing between endometriosis-correlated ovarian carcinoma, in which transitional lesions support a histopathologic progression from endometriosis to carcinoma, and endometriosis-incidental ovarian carcinoma, in which endometriosis is present but no transitional lesions are identified [11]. The specific risk profiles to guide individual counseling of patients with endometriosis have yet to be clearly defined and the prognostic implications of concurrent endometriosis at the time of ovarian cancer diagnosis remain unclear. Therefore, we conducted this study to further evaluate whether endometriosis is an independent prognostic factor for survival in patients with ovarian cancer and concurrent endometriosis.

## Methods

### Study design

This retrospective cohort study evaluated survival outcomes and clinical characteristics of patients with histologically confirmed primary malignant ovarian neoplasms or borderline ovarian tumors, with or without diagnosed concurrent endometriosis at the time of primary treatment. The study was conducted at Charité – Universitätsmedizin Berlin, a German tertiary academic medical center and certified European Society of Gynecological Oncology (ESGO) Centre of Excellence. The study was approved by the local institutional ethics committee (EA2/266/22). Due to the retrospective nature of the analysis, written informed consent was not obtained from the patients included in this study.

### Participants and data collection

Eligible were patients who received primary treatment for a malignant or borderline ovarian tumor at the department of gynecology of the Charité – Universitätsmedizin Berlin between January 1, 2014, and July 31, 2025. Exclusion criteria included primary surgery performed at another institution, ovarian metastases from non-ovarian malignancies, or incomplete clinicopathologic or follow-up data. There were no restrictions regarding histologic subtype or primary treatment strategy. Patients were identified through the institutional cancer registry. Clinical, pathological, and treatment data were extracted from electronic medical records and the institutional pathology database. Follow-up information, including survival status, was obtained from the institutional cancer registry.

### Classification of endometriosis status

The diagnosis of concurrent endometriosis was defined as histologic confirmation of adenomyosis uteri and/or ovarian and/or peritoneal endometriotic lesions identified during surgery for a malignant or borderline ovarian tumor. Patients with endometriosis-associated ovarian tumors were further subclassified based on the classification proposed by Mezzapesa [11]:

1. *Endometriosis-correlated ovarian tumors*, defined by the evidence of a direct histopathologic transition from an endometriosis lesion to the tumor and/or proof of transitional lesions; or.
2. *Endometriosis-incidental tumors*, defined as endometriosis identified at primary treatment without histologic proof of direct transition or proof of transitional lesions.

The reference cohort included patients with malignant or borderline ovarian tumors without histologically confirmed endometriosis and was age-adjusted to the endometriosis cohort using a 1:5 ratio.

### Outcomes and statistical analysis

The primary outcome was overall survival, defined as the time from diagnosis to death from any cause or last follow-up. Secondary outcomes included disease-free survival and clinicopathological characteristics of patients with endometriosis associated tumors. Disease-free survival was defined as the time from definitive surgery to the first occurrence of disease recurrence or death from any cause. Continuous variables are presented as mean (standard deviation) or median (interquartile range), depending on scale; categorical variables are reported as counts and percentages. Group comparisons were performed using the Pearson χ² test for categorical variables and one-way analysis of variance (ANOVA) for continuous variables. Survival analyses were restricted to patients with epithelial ovarian carcinoma. Survival estimates were performed with the use of the Kaplan-Meier method and compared using log-rank testing. Multivariable Cox proportional hazards models were used to estimate hazard ratios (HRs) and 95% confidence intervals (CIs) for overall survival, adjusting for age, histologic subtype, FIGO stage, endometriosis status, and complete macroscopic tumor resection. All statistical tests were two-sided, with α < 0.05 considered statistically significant. Analyses were conducted using IBM SPSS Statistics, version 31.0.0 (IBM Corp., Armonk, NY, USA). In accordance with the journal’s guidelines, we will provide our data for independent analysis by a selected team by the Editorial Team for the purposes of additional data analysis or for the reproducibility of this study in other centers if such is requested.

## Results

### Patient population

Between January 2014 and July 2025, 2,164 patients treated for primary malignant or borderline ovarian tumors at the Charité – Universitätsmedizin Berlin met the eligibility criteria. Among these, 176 patients (8.1%) had histologically confirmed endometriosis identified during primary surgery. Endometriosis-correlated ovarian tumors, defined by a direct histopathologic transition between endometriosis and the tumor, were identified in 22 patients (1.0%). In the remaining 154 patients (7.1%), endometriosis was present without histologic evidence of a direct transition to a malignant or borderline ovarian tumor; these cases were classified as endometriosis-incidental ovarian tumors. The average age of the overall cohort was 56.4 ± 14.3 years. Patients with endometriosis were significantly younger than those without endometriosis (mean age, 48.6 ± 11.8 years vs. 57.1 ± 14.4 years; *p* <.001). To adjust for this age difference, the reference cohort of patients without endometriosis was age-matched at a 1:5 ratio and included 880 individuals. Baseline characteristics of the overall population are presented in Table S1.

### Clinical characteristics

Tumor type differed significantly among subgroups (*p* <.001). Epithelial ovarian carcinoma was more frequent in patients with endometriosis-correlated ovarian tumors (86.4%) than in patients with endometriosis-incidental ovarian tumors (60.4%) or without endometriosis (75.8%), whereas borderline ovarian tumors were more often diagnosed in patients with endometriosis-incidental ovarian tumors (31.8%) compared to patients without endometriosis (19.0%) or with endometriosis-correlated ovarian tumors (9.1%; *p* <.001). Patients with endometriosis-correlated ovarian tumors and endometriosis-incidental ovarian tumors were usually diagnosed in early disease stages (FIGO I–II), with 86.4% of endometriosis-correlated ovarian tumors diagnosed in stage I. In contrast, patients without endometriosis presented in 59% of cases with advanced-stage disease (FIGO III–IV; *P* <.001).

Eastern Cooperative Oncology Group (ECOG) performance status was similar across subgroups, with most patients having a good functional status. Most patients in all subgroups underwent primary surgery. Macroscopic complete resection was achieved in the majority of cases without significant intergroup differences. Detailed results for the baseline characteristics are shown in Table 1.

**Table 1:** Characteristics of patients with endometriosis-incidental tumors, endometriosis-correlated tumors, and an age-matched reference cohort without evidence of endometriosis

Patient Characteristics		All (*n* = 1056)	Endometriosis- incidental (*n* = 154)	Endometriosis - correlated (*n* = 22)	No Proof of Endometriosis (*n* = 880)
Age (years) Mean ± SD		48.7 ± 11.9	48.5 ± 11.9	49.0 ± 10.9	48.7 ± 11.9
Epithelial Ovarian Cancer		778 (73.7)	92 (60.4)	19 (86.4)	667 (75.8)
	*Serous HG*	*509/778 (65.4)*	*43/92 (46.7)*	*0/19 (0)*	*466/667 (69.9)*
	*Serous LG*	*73/778 (9.4)*	*9/92 (9.8)*	*1/19 (5.3)*	*63/667 (9.4)*
	*Endometrioid LG*	*74/778 (9.5)*	*18/92 (19.6)*	*8/19 (42.1)*	*48/667 (7.2)*
	*Endometrioid HG*	*22/778 (2.8)*	*5/92 (5.4)*	*2/19 (10.5)*	*15/667 (2.2)*
	*Clear cell*	*43/778 (5.5)*	*8/92 (8.7)*	*8/19 (42.1)*	*27/667 (4.0)*
	*Other*	*5/778 (7.4)*	*10/92 (10.9)*	*0/19 (0)*	*48/667 (7.2)*
Borderline		278 (26.3)	49 (31.8)	2 (9.1)	167 (19.0)
	*Serous*	*14/278 (50.7)*	*35/49 (71.4)*	*1/2 (50.0)*	*105/167 (62.9)*
	*Mucinous*	*73/278 (26.3)*	*14/49 (28.6)*	*0*	*59/167 (35.3)*
	*Endometrioid*	*5/278 (1.8)*	*1/49 (2.0)*	*1/2 (50.0)*	*3/167 (1.8)*
Sex Cord		38 (3.6)	7 (4.5)	0	31 (3.5)
Germ Cell		18 (1.7)	4 (2.6)	0	14 (1.6)
Sarcoma		3 (0.3)	1 (0.6)	1 (4.5)	1 (0.1)
Stage	1	352 (33.3)	76 (49.4)	19 (86.4)	257 (29.2)
	2	84 (8.0)	17 (11.0)	0	67 (7.6)
	3	432 (40.9)	32 (20.8)	2 (9.1)	398 (45.2)
	4	138 (13.1)	17 (11.0)	0	121 (13.8)
ECOG Status	0	797 (75.5)	117 (76.0)	20 (95.2)	660 (75.0)
	1	190 (17.9)	26 (16.9)	2 (4.8)	162 (18.4)
	2	2 (1.9)	0 (0)	0 (0)	2 (0.9)
Surgery	Primary	969 (91.9)	147 (95.5)	22 (100)	800 (90.9)
	Interval	87 (8.1)	7 (4.5)	0	80 (9.1)
Complete Resection		915 (86.6)	142 (92.2)	22 (100)	751 (85.3)

*Abbreviations ECOG* Eastern Cooperative Oncology Group, *HG* high-grade, *LG * low-grade, *SD *standard deviation

The localization of endometriosis differed significantly between patients with endometriosis-correlated ovarian tumors and those with endometriosis-incidental ovarian tumors: Ovarian endometriosis was more frequent in patients with endometriosis-correlated ovarian tumors (20 [90.9%]) than in patients with endometriosis-incidental ovarian tumors (38 [24.7%]; *p* <.001), Table S2. No significant difference was observed for peritoneal endometriosis, which was reported in 11 (50.0%) and 59 (38.3%) patients, respectively. 59 patients had Adenomyosis without proof of peritoneal or ovarian endometriosis. Adenomyosis uteri was diagnosed in 77 (50.0%) patients with endometriosis-incidental ovarian tumors but in only 4 (18.2%) patients with endometriosis-correlated ovarian tumors.

Most patients had no prior diagnosis of endometriosis, and rates did not differ significantly between subgroups (20 [13.0%] in endometriosis-incidental ovarian tumors vs. 4 [18.2%] in endometriosis-correlated ovarian tumors; *p* >.05). Patients with a history of endometriosis were younger, had lower ECOG performance status, and were less likely diagnosed with high-grade serous ovarian cancer. In contrast, p53 mutation status, BRCA mutation status, tumor grade, surgical approach, and tumor stage did not differ between patients with and without previously diagnosed endometriosis. For detailed information, please refer to Supplementary Table S3.

Menopausal status and reproductive history were also similar between groups. Most patients were premenopausal (92 [59.7%] in endometriosis-incidental ovarian tumors and 16 [72.7%] in endometriosis-correlated ovarian tumors). The number of pregnancies and live births did not differ significantly. Patients with endometriosis-correlated ovarian tumors had a mean of 0.7 ± 0.8 pregnancies and 0.5 ± 0.7 live births, compared with 1.1 ± 1.4 pregnancies and 0.8 ± 1.1 live births in those with endometriosis-incidental ovarian tumors. Nulligravidity was observed in 54 (35.1%) patients with endometriosis-incidental ovarian tumors and 10 (45.5%) with endometriosis-correlated ovarian tumors, and nulliparity in 64 (41.6%) and 11 (50.0%) patients, respectively. Detailed results are shown in Table S2.

### Histopathological characteristics

Histologic subtypes of epithelial ovarian carcinoma varied significantly among subgroups (*p* <.001). High-grade serous carcinoma was the most frequent subtype in patients without endometriosis (69.9%) and in nearly half of those with endometriosis-incidental ovarian tumors (46.2%), whereas endometrioid (52.6%) and clear cell (42.1%) histology was most common in the endometriosis-correlated ovarian tumors group. Rates of low-grade serous carcinoma were comparable between groups (Table 1).

Among patients with epithelial ovarian carcinoma, tumor grade differed significantly between patients with and without concurrent endometriosis (34.2% vs. 20.8%, *p* =.002). Patients with endometriosis had a higher proportion of low-grade tumors (G1–2). However, no significant difference in tumor grade was observed between endometriosis-associated ovarian carcinoma and endometriosis-incidental ovarian carcinoma (Table 2). Expression of estrogen and progesterone receptors did not differ significantly between endometriosis subgroups, with most tumors showing estrogen receptor positivity greater than 10% and approximately half demonstrating progesterone receptor expression greater than 10%. Notably, no patient with endometriosis-correlated ovarian carcinoma showed a p53 mutational pattern, whereas such alterations were identified in about one-third of endometriosis-incidental ovarian carcinomas. In line with these findings, no BRCA1/2 mutations were reported in the endometriosis-correlated ovarian carcinoma cohort, compared with 14.3% in endometriosis-incidental ovarian carcinoma. Detailed results are presented in Table 2.

**Table 2:** Histopathological characteristics of patients with endometriosis and epithelial ovarian cancer, categorized into endometriosis-incidental ovarian cancer and endometriosis-associated ovarian cancer

Tumor Characteristics				All Endometriosis (*n* = 111)	Endometriosis-incidental (*n* = 92)	Endometriosis-correlated (*n* = 19)	Alpha
Grading	*Low-grade (G1-G2)*			*38/111 (34.2)*	*29/92 (31.5)*	*9/19 (47.4)*	*n.s.*
	*High-grade (G3)*			*70/111 (63.1)*	*60/92 (65.2)*	*10/19 (52.6)*	
	*Gx*			*4/111 (3.6)*	*4/92 (4.3)*	*0/19 (0)*	
P53 Mutation				*29/87 (33.3)*	*29/75 (38.7)*	*0/12 (0)*	*< 0.001*
ER		*0*		*27/87 (31.0)*	*21/72 (29.2)*	*6/15 (40.0)*	*n.s.*
		*1–10%*		*9/87 (10.3)*	*9/72 (12.5)*	*0/15 (0)*	
		*11–100%*		*5/87 (58.6)*	*42/72 (45.7)*	*9/15 (60.0)*	
PR			*0*	*44/81 (54.3)*	*36/66 (54.5)*	*8/15 (53.3)*	*n.s.*
			*1–10%*	*6/81 (7.4)*	*6/66 (9.1)*	*0/15 (0)*	
			*11–100%*	*3/81 (38.2)*	*24/66 (36.4)*	*7/15 (46.7)*	
BRCA1/2 Mutation				*7/59 (11.8)*	*7/49 (14.3)*	*0/10 (0)*	*n.s.*
Deficient MMR				*1/62 (1.6)*	*0/52 (0)*	*1/10 (10.0)*	*n.s.*

Data completeness varied, and denominators reflect the number of tumors with available results. Intergroup comparisons between patients with endometriosis-incidental and endometriosis-associated ovarian cancer were performed using the Pearson χ² test

*Abbreviations MMR* mismatch repair, *n.s.* non-significant

In a subsequent analysis, patients with endometriosis (including both incidental and associated tumors; *n* = 117) were compared with patients diagnosed with adenomyosis without concurrent endometriosis (*n* = 59). Patients with adenomyosis were generally older, less frequently premenopausal, and more likely to have ECOG performance status of 1. They more commonly presented with high-grade serous ovarian cancer and were more often diagnosed at advanced disease stages. In contrast, the prevalence of low-grade endometrioid and borderline ovarian tumors was lower in this group. Consistent with these findings, rates of p53 mutation and high tumor grade were higher among patients with adenomyosis. Detailed results are provided in Supplementary Table S4.

### Survival analysis

**Fig. 1:**
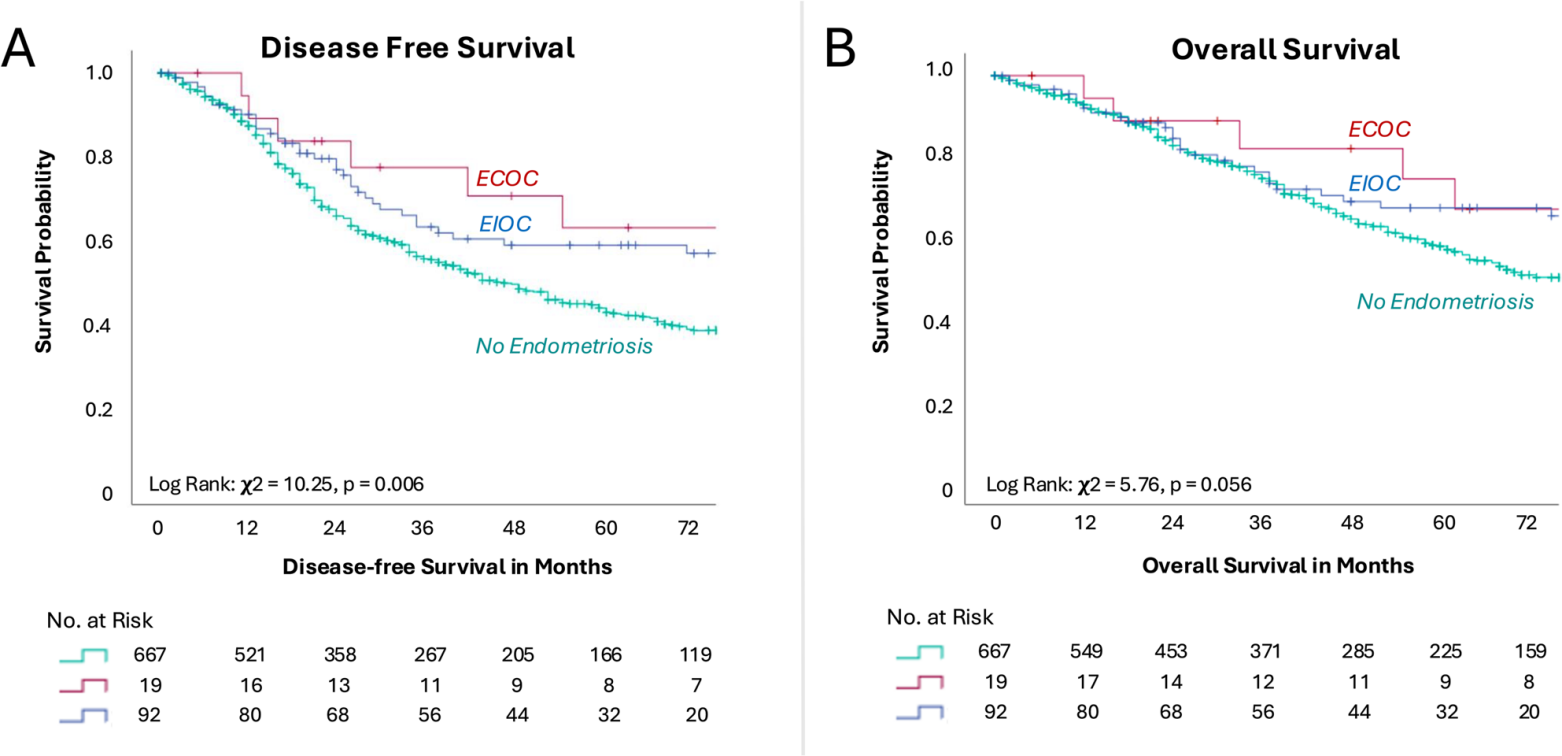
Kaplan–Meier Estimates of (**A**) Disease-Free Survival and (**B**) Overall Survival in Patients With and Without Endometriosis. Green lines indicate patients without endometriosis, blue lines patients with endometriosis-incidental ovarian cancer (EIOC), and red lines patients with endometriosis-correlated ovarian cancer (ECOC). Tick marks represent censored observations. Numbers of patients at risk are shown below the corresponding time points on the x-axis

Only patients with epithelial ovarian cancer were included in the survival analysis. As of the clinical cutoff date (July 2025), the median follow-up for patients with epithelial ovarian cancer was 41 months (95% CI, 4–124.05). The median overall survival for patients without endometriosis was 79 months (95% CI, 64.3–93.7). The median overall survival for patients with endometriosis-incidental ovarian carcinoma and endometriosis-correlated ovarian carcinoma had not been reached. Kaplan–Meier estimates for overall survival showed no statistically significant differences between subgroups (Fig. 1). The stratified hazard ratio (HR) for overall survival was 2.07 (95% CI, 0.82–5.25) for patients with endometriosis-correlated ovarian carcinoma and 1.01 (95% CI, 0.68–1.52) for those with endometriosis-incidental ovarian carcinoma.

Multivariate Cox regression for overall survival analysis identified macroscopic complete resection, high-grade serous, low-grade serous, and endometrioid histology as predictors of favorable outcomes, whereas advanced disease stage and older age were associated with worse outcomes. The diagnosis of endometriosis-incidental ovarian carcinoma and endometriosis-correlated ovarian carcinoma was not an independent predictor of overall survival (Table 3).

**Table 3:** Multivariate Cox Regression Analysis for Overall Survival for Patients with Epithelial Ovarian Cancer

	Overall Survival	
	HR (95%CI)	*p*
Endometriosis-incidental	*1.02 (0.68–1.52)*	*n.s.*
Endometriosis-correlated	*2.07 (0.82–5.25)*	*n.s.*
High-grade Serous	*0.44 (0.28–0.70)*	*< 0.001*
Low-grade Serous	*0.22 (0.12–0.41)*	*< 0.001*
Endometrioid	*0.39 (0.20–0.77)*	*0.006*
Clear Cell Histology	*0.71 (0.45–0.88)*	*n.s.*
Stage	*1.95 (1.66–2.30)*	*< 0.001*
Macroscopic Complete Resection	*0.499 (0.38–0.65)*	*< 0.001*

*Abbreviations HR *hazard ratio*, n.s. *non-significant

Median time to disease progression or death was 44 months (95% CI, 36.79–51.21) for patients without endometriosis. Median disease-free survival for patients with endometriosis-incidental ovarian carcinoma and endometriosis-correlated ovarian carcinoma was not reached. Kaplan–Meier estimates for disease-free survival differed significantly between subgroups, (Fig. 1). But stratified HR for disease progression or death did not reach significance, with HR 1.86 (95% CI, 0.76–4.56) for endometriosis-correlated ovarian carcinoma and 1.07 (95% CI, 0.75–1.53) for endometriosis-incidental ovarian carcinoma.

## Discussion

In this large, single-center retrospective study from a high-volume ESGO-accredited academic institution, patients with primary malignant or borderline ovarian tumors and histologically confirmed concurrent endometriosis were diagnosed at younger age and with more favorable clinicopathologic characteristics than those without endometriosis. Patients with endometriosis more often presented with early FIGO stage disease, low tumor grade, and endometrioid or clear-cell histotypes. The majority of endometriosis-associated ovarian tumors were subclassified as endometriosis-incidental tumors, in which endometriosis was present at diagnosis of ovarian cancer without evidence of transition to the tumor. Proof of transition (endometriosis-correlated) was only identified in 12.5% of cases.

Ovarian endometriosis was more common in endometriosis-correlated tumors than in endometriosis-incidental tumors, while history of endometriosis, menopausal status and reproductive history were comparable between subgroups. In endometriosis-correlated ovarian carcinoma no p53 mutation or BRCA1/2 alterations were identified, while in endometriosis-incidental ovarian carcinoma p53 aberrations and BRCA1/2 mutations were seen in some patients. Most patients in both subgroups underwent primary surgery with high macroscopic complete resection rates. After adjustment for age, stage, histotype, and cytoreduction, endometriosis-correlated and endometriosis-incidental ovarian carcinoma were not independent predictors for overall and disease-free survival. Histology, age, complete cytoreduction and disease stage remained the strongest predictors of survival.

Notably, the stage distribution of the overall cohort was shifted toward earlier tumor stages, with more than 40% of cases presenting at stage I/II. This pattern may be largely explained by the applied age adjustment and the resulting younger overall cohort (mean age, 48.7 years), as several studies have reported higher rates of early-stage disease in younger patient populations [21–23]. In line with previous data [6, 10, 11], endometriosis-associated ovarian carcinomas were to a high proportion of clear cell or endometrioid histology. Epidemiologic studies reported an approximately 2- to 4-fold increased risk of clear cell and endometrioid ovarian carcinoma and little or no association with high-grade serous or mucinous ovarian cancer in patients with endometriosis [6, 8, 24]. High-grade serous carcinomas are almost universally characterized by p53 mutations [25, 26], whereas endometrioid and clear cell ovarian cancers typically show distinct mutational profiles, including alterations in PTEN, PIK3CA, and ARID1A, and exhibit less frequently p53 mutations [26–30].

Similarly, in endometriosis-associated ovarian cancer, ARID1A, PIK3CA, and PTEN mutations and activation of the PI3K/AKT/mTOR pathway have been described as early and defining events of tumor development [18, 30–32]. Notably, some of these genomic alterations have been also described in patients with deep infiltrating endometriosis without cancer [19]. Consistent with this molecular pattern, endometriosis-correlated carcinomas in our cohort showed an absence of p*53* mutation. Germline BRCA1/2 mutations, which primarily predispose to high-grade serous carcinoma [33], were also absent in the endometriosis-correlated cohort. The high proportion of low-grade and endometrioid or clear cell tumors within the endometriosis subgroups further supports the concept of a type I, non-p53-driven tumorigenic pathway for endometriosis-correlated ovarian cancer [4–6, 10, 11, 34].

As endometriosis and adenomyosis are considered related entities within a shared disease spectrum, with overlapping pathophysiological mechanisms [35, 36], a similar distribution of ovarian cancer histological subtypes might be expected. However, patients with adenomyosis without concurrent endometriosis presented in our subgroup analysis with a clinical profile more resembling that of patients without endometriosis. Specifically, these patients showed higher rates of high-grade serous ovarian cancer, a greater frequency of p53-mutated tumors, a lower prevalence of type I ovarian cancers, and presented more frequently at advanced tumor stages.

These findings contrast with a Dutch population-based study, which reported an increased incidence of ovarian cancer for both patients with endometriosis and adenomyosis, with the strongest associations observed for endometrioid and clear cell subtypes in the adenomyosis cohort [37]. Notably, most patients with adenomyosis in that study had isolated adenomyosis without concurrent endometriosis. Further prospective evidence is needed to clarify whether adenomyosis in the absence of endometriosis is associated with the development of type I ovarian cancer to a similar extent as endometriosis.

In the present cohort, most patients with endometriosis-correlated or incidental ovarian cancer had no documented prior diagnosis of endometriosis. This finding likely reflects the well-reported diagnostic gap of endometriosis, as population-based estimates suggest that endometriosis affects approximately 5–10% of individuals of reproductive age [38], whereas reported prevalence in routine clinical data is substantially lower, ranging only up to 2% [39, 40]. Beyond general underdiagnosis, undiagnosed and consequently untreated endometriosis may constitute an independent risk factor for the development of endometriosis-associated ovarian cancer itself. In the absence of appropriate surgical and endocrine management, malignant transformation could be promoted within the chronic inflammatory, iron-rich, oxidative, and estrogen-driven microenvironment of persisting endometriotic lesions, particularly within ovarian endometriomas. Given the observational design of this study and the lack of data on prior endometriosis treatment, this hypothesis remains highly speculative. Prospective studies evaluating the impact and quality of endometriosis management on cancer risk are needed.

Endometriosis-correlated carcinoma with evidence of transition from endometriosis to ovarian carcinoma was identified primarily in early-stage disease, consistent with a 2014 meta-analyses reporting that endometriosis-associated ovarian cancers are more frequently diagnosed in younger patients and at earlier FIGO stages and with lower tumor grades [4]. Several factors may be attributed to this high proportion of early-stage diagnoses among patients with endometriosis-associated tumors. The symptomatic nature of endometriosis might lead to earlier clinical evaluation and diagnosis of pelvic masses. Tumor biology may also play a substantial role, as high-grade serous carcinomas show rapid growth kinetics [41], whereas type I ovarian cancers are more slow growing tumors and more frequently diagnosed in early stages [42]. The paucity of advanced endometriosis-correlated ovarian cancer may also reflect challenges in histopathologic diagnosis. In advanced ovarian cancer, extensive tumor overgrowth and necrosis might obscure precursor lesions, making it difficult to identify a direct histologic transition according to the Sampson and Scott criteria or endometriosis lesions at all [20].

No difference in overall and disease-free survival was observed between patients with endometriosis-correlated or endometriosis-incidental ovarian cancer and those without endometriosis. Kaplan–Meier estimates showed excellent survival outcomes in both endometriosis subgroups, with median overall survival not reached at last follow-up. Similar findings have been reported in multicenter and population-based studies [43]. After adjustment for age, stage, histologic subtype, and surgical cytoreduction, endometriosis was not an independent prognostic factor for overall survival. This finding aligns with a large meta-analysis by Kim and colleagues, which reported that although unstratified overall survival appeared better in ovarian cancer patients with endometriosis, the survival difference disappeared after stratifying by stage and tumor characteristics [4].

The favorable outcomes associated with endometriosis associated ovarian cancer may therefore largely be attributable to its favorable clinical profile (younger age, early stage, low-grade histology) rather than a protective effect of endometriosis itself. Consistent with this, our study confirmed that well-known prognostic factors such as FIGO stage and macroscopic complete resection remained the strongest predictors for favorable survival outcomes [44–46].

On the other hand, rare and occasionally aggressive histologic subtypes developing in the setting of endometriosis have been previously reported. Mezzapesa and colleagues recently described a series of endometriosis-associated mesonephric-like ovarian adenocarcinomas [47], highlighting that malignant transformation in endometriosis is not only limited to endometrioid and clear cell histotypes. In the present cohort, no rare high-grade tumors were identified. This finding should be interpreted with caution, as it likely reflects both the overall rarity of these entities and the limited sample size of our cohort rather than evidence against their occurrence in endometriosis-associated disease.

Notably, two cases of low-grade endometrial stromal sarcoma were observed in our analysis, with one case each in the endometriosis-incidental and endometriosis-correlated cohort. Although uncommon, the association between extrauterine low-grade endometrial stromal sarcoma and endometriosis has been reported previously [48], supporting the concept that endometriosis may serve as a precursor niche for a broader spectrum of gynecologic neoplasms than is generally appreciated.

### Strength and limitations

This study has several limitations. Its retrospective design bears the risk for residual confounding and selection bias. In our cohort, only 22 patients (1.0% of the overall population) were classified as having endometriosis-correlated tumors. This proportion is markedly lower than the 28.24% reported by Mezzapesa et al. [11]. The frequency of endometriosis-correlated tumors in our series was approximately sevenfold higher than that of endometriosis-incidental cases, whereas similar rates were reported in the Mezzapesa publication. These differences may be explained by methodological factors, including the retrospective unicentric design of the present study and potential underdiagnosis or underreporting of endometriosis correlated ovarian cancer in routine clinical practice. The small number of patients with endometriosis-correlated tumors may limit the statistical power of the subgroup analyses. Consequently, the multivariable survival analysis may be underpowered, and the potential prognostic impact of endometriosis-associated ovarian cancer may be underestimated.

Immunohistochemistry and molecular pathology were not uniformly performed across all cases. Clinical information on prior endocrine treatment, the individual symptomatic burden of endometriosis, and the quality of endometriosis treatment was not assessed, as these data were not consistently documented in the patient records. Key strengths of this study include the large cohort of patients with primary malignant and borderline ovarian tumors and the comprehensive characterization of both clinical and histopathologic variables. The analysis was based on consecutively treated patients at a high-volume, ESGO-accredited tertiary referral center, with standardized diagnostic workup, surgical management, and follow-up.

## Conclusions

In conclusion, ovarian tumors correlated with endometriosis represent a clinically relevant entity characterized by an earlier age at diagnosis, an earlier disease stage and endometrioid or clear cell histology. In this analysis endometriosis itself did not independently determine survival. However, these findings should be interpreted with caution given the relatively small number of patients with endometriosis-correlated ovarian cancer in our cohort. At present, management of endometriosis-associated ovarian cancer should follow established international ovarian cancer guidelines.

There remains a clear clinical need for prospective registries and larger, well-annotated cohorts to better define risk profiles and to more precisely delineate the prognostic impact of endometriosis-associated ovarian cancer. Beyond comprehensive clinical data, including quality of prior endometriosis management, future studies should evaluate molecular tumor characteristics, such as ARID1A, PIK3CA, PTEN, and mismatch-repair profiles to further clarify their role in endometriosis-associated carcinogenesis. Integrating these data may enable more refined risk stratification in patients with endometriosis and support the development of tailored management strategies for this distinct ovarian cancer population.

## Supplementary Information

Supplementary Material 1.

Supplementary Material 2.

Supplementary Material 3.

Supplementary Material 4.

## Data Availability

The datasets used and/or analysed during the current study are available from the corresponding author on reasonable request.
