# Supplementary material for: Characteristics and outcomes of patients with endometriosis and malignant or borderline ovarian tumors: real-world evidence from an ESGO centre of excellence
Source: BMC Cancer. 2026 Apr 16;26:495. doi: 10.1186/s12885-026-15980-w (PMC13088446; doi:10.1186/s12885-026-15980-w)
Supplement: Supplementary file 1 — Supplementary Material 1. [file 12885_2026_15980_MOESM1_ESM.pdf]

Table S1 - Patient Characteristics for the Overall Population

Patient Characteristics		Endometriosis-incidental (n=154)	Endometriosis - correlated (n=22)	Age-adjusted Control (n=880)	Overall (n=2165)
**Age (years)**	Mean ± SD	**48.5 ± 11.9**	**49.0 ± 10.9**	**48.7 ± 11.9**	**57 (47-67)**
**Epithelial Ovarian Cancer**		**93 (60.4)**	**19 (86.4)**	**667 (75.8)**	**1728 (79.8)**
	Serous HG	*43/93 (46.2)*	*0/19 (0)*	*466/667 (69.9)*	*1248/1728 (72.2)*
	Serous LG	*9/93 (9.7)*	*1/19 (5.3)*	*63/667 (9.4)*	*134/1728 (7.8)*
	Endometrioid LG	*18/93 (19.4)*	*8/19 (42.1)*	*48/667 (7.2)*	*136/1728 (7.8)*
	Endometrioid HG	*5/93 (5.3)*	*2/19 (10.5)*	*15/667 (2.2)*	*70/1728 (4.1)*
	Clear cell	*8/93 (8.6)*	*8/19 (42.1)*	*27/667 (4.0)*	*8/1728 (5.2.)*
	Other	*10/93 (10.8)*	*0/19 (0)*	*48/667 (7.2)*	*46/1728 (2.7)*
**Borderline**		**49 (31.8)**	**2 (9.1)**	**167 (19.0)**	**345 (15.9)**
	Serous	*35/49 (71.4)*	*1/2 (50.0)*	*105/167 (62.9)*	*223/345 (64.6)*
	Mucinous	*14/49 (28.6)*	*0*	*59/167 (35.3)*	*113/345 (32.8)*
	Endometrioid	*1/49 (2.0)*	*1/2 (50.0)*	*3/167 (1.8)*	*9/345 (2.6)*
**Sex Cord**		**7 (4.5)**	**0**	**31 (3.5)**	**56 (2.6)**
**Germ Cell**		**4 (2.6)**	**0**	**14 (1.6)**	**29 (1.3)**
**Sarcoma**		**1 (0.6)**	**1 (4.5)**	**1 (0.1)**	**7 (0.3)**
**Stage**	**1**	**76 (49.4)**	**19 (86.4)**	**257 (29.2)**	**592 (28.4)**
	**2**	**17 (11.0)**	**0**	**67 (7.6)**	**152 (7.3)**
	**3**	**32 (20.8)**	**2 (9.1)**	**398 (45.2)**	**999 (47.9)**
	**4**	**17 (11.0)**	**0**	**121 (13.8)**	**341 (16.4)**
**ECOG**	**0**	**117 (76.0)**	**20 (95.2)**	**660 (75.0)**	**1504 (73.9)**
	**1**	**26 (16.9)**	**2 (4.8)**	**162 (18.4)**	**483 (23.7)**
	**2**	**0 (0)**	**0 (0)**	**2 (0.9)**	**46 (2.1)**
**Surgical Approach**	**Primary**	**147 (95.5)**	**22 (100)**	**800 (90.9)**	**1 (0.1)**
	**Interval**	**7 (4.5)**	**0**	**80 (9.1)**	**2 (0.1)**
**Complete Resection**	**yes**	**142 (92.2)**	**22 (100)**	**751 (85.3)**	**1953 (90.2)**
