# Supplementary material for: Characteristics and outcomes of patients with endometriosis and malignant or borderline ovarian tumors: real-world evidence from an ESGO centre of excellence
Source: BMC Cancer. 2026 Apr 16;26:495. doi: 10.1186/s12885-026-15980-w (PMC13088446; doi:10.1186/s12885-026-15980-w)
Supplement: Supplementary file 2 — Supplementary Material 2. [file 12885_2026_15980_MOESM2_ESM.pdf]

Table S2: Clinical Characteristics of patients with simultaneous endometriosis and patients with endometriosis associated ovarian tumors. Results are presented as absolute numbers (percentage).

**Patient Characteristics**	**Simultaneous Endometriosis (n= 154)**	**Endometriosis associated Tumor (n= 22)**	**Alpha**
**Current Proof of Endometriosis**			
Ovarian	*38 (24.7)*	*20 (90.9)*	*<0.001*
Peritoneal	*59 (38.3)*	*11 (50.0)*	*n.s.*
Adenomyosis	*77 (50.0)*	*4 (8.2)*	*0.008*
**History of Endometriosis**	*20 (13.0)*	*4 (18.2)*	*n.s.*
**Menopausal State**			*n.s.*
Premenopausal	*92 (59.7)*	*16 (72.7)*	
Postmenopausal	*62 (40.3)*	*6 (26.3)*	
**Number of Pregnancies**			*n.s.*
0	*54 (35.1)*	*10 (45.5)*	
1	*29 (18.8)*	*4 (18.2)*	
≥2	*38 (24.7)*	*4 (18.2)*	
missing	*33 (21.4)*	*4 (18.2)*	
**Number of Childbirths**			*n.s.*
0	*64 (41.6)*	*11 (50.0)*	
1	*29 (18.8)*	*6 (27.3)*	
≥2	*29 (18.8)*	*2 (9.1)*	
missing	*32 (20.8)*	*3 (13.6)*	
