# Supplementary material for: Characteristics and outcomes of patients with endometriosis and malignant or borderline ovarian tumors: real-world evidence from an ESGO centre of excellence
Source: BMC Cancer. 2026 Apr 16;26:495. doi: 10.1186/s12885-026-15980-w (PMC13088446; doi:10.1186/s12885-026-15980-w)
Supplement: Supplementary file 3 — Supplementary Material 3. [file 12885_2026_15980_MOESM3_ESM.pdf]

Supplementary Table S3. Characteristics of patients with previous diagnosed endometriosis and patients with endometriosis and no history of endometriosis

Patient Characteristics		History of Endometriosis (n=24)	No History of Endometriosis (n=152)	Alpha
**Age (years)** Mean ± SD		42.6 ± 10.6	49.5 ± 11.8	*.007*
**Premenopausal**		6 (37.5)	62 (40.8)	*.140*
**Epithelial Ovarian Cancer**		14 (58.3)	97 (63.8)	*.701*
	Serous HG	2 (8.3)	41 (27.0)	*.048*
	Serous LG	1 (4.2)	9 (5.9)	*.730*
	Endometrioid LG	6 (25.0)	20 (13.2)	*.129*
	Endometrioid HG	1 (4.2)	6 (3.9)	*.959*
	Clear cell	3 (12.5)	13 (8.6)	*.532*
**Borderline**		8 (33.3)	44 (28.9)	*.638*
**Stage**	**1**	18 (75.0)	77 (50.7)	*.190*
	**2**	2 (8.3)	15 (9.9)	
	**3**	2 (8.3)	32 (21.1)	
	**4**	1 (4.2)	16 (10.5)	
**ECOG Status**	**0**	22 (91.7)	115 (75.7)	*.025*
	**1**	0	27 (17.8)	
**Surgery**	**Primary**	23 (95.8)	146 (32.3)	*.959*
	**Interval**	1 (4.2)	6 (39.5)	
**Complete Resection**		22 (91.7)	142 (93.4)	*.751*
**Grading**	*G1-G2*	9 (37.5)	32 (21.1)	*.064*
	G3	7 (29.2)	67 (44.1)	
	Gx	8 (33.3)	53 (34.9)	
**p53 ***		3 (25.0)	43 (50.6)	*.097*
**BRCA ***		1 (14.3)	6 (11.1)	*.804*

*Intergroup comparisons between patients with endometriosis and previous diagnosis of endometriosis and no previous history were performed using the Pearson χ^2 test for categorial variables and independent t-test for continuous variables. Abbreviations: ECOG = Eastern Cooperative Oncology Group; HG = high-grade; LG = low-grade; SD = standard deviation. * relative percentages.*
