# Supplementary material for: Characteristics and outcomes of patients with endometriosis and malignant or borderline ovarian tumors: real-world evidence from an ESGO centre of excellence
Source: BMC Cancer. 2026 Apr 16;26:495. doi: 10.1186/s12885-026-15980-w (PMC13088446; doi:10.1186/s12885-026-15980-w)
Supplement: Supplementary file 4 — Supplementary Material 4. [file 12885_2026_15980_MOESM4_ESM.pdf]

Supplementary Table S4. Characteristics of patients with endometriosis and patients with adenomyosis.

**Patient Characteristics**		**Adenomyosis only (n=59)**	**Endometriosis (n=117)**	**Alpha**
**Age (years)** Mean ± SD		53.5 ± 9.7	46.0 ± 12.0	*<.001*
**Premenopausal**		29 (49.2)	79 (67.5)	*.018*
**Epithelial Ovarian Cancer**		41 (69.5)	70 (59.8)	*.210*
	Serous HG	25 (42.4)	18 (15.4)	*<.001*
	Serous LG	3 (5.1)	7 (6.0)	*.808*
	Endometrioid LG	4 (6.8)	22 (18.8)	*.034*
	Endometrioid HG	2 (3.4)	5 (4.3)	*.777*
	Clear cell	2 (3.4)	14 (12.0)	*.062*
**Borderline**		11 (18.6)	41 (35.0)	*.024*
**Stage**	**1**	20 (33.9)	75 (64.1)	*<.001*
	**2**	4 (6.8)	13 (11.1)	
	**3**	20 (33.9)	14 (12.0)	
	**4**	12 (20.3)	5 (4.3)	
**ECOG Status**	**0**	37 (62.7)	100 (85.5)	*<.001*
	**1**	18 (30.5)	9 (7.7)	
**Surgery**	**Primary**	54 (91.5)	115 (98.3)	*.030*
	**Interval**	5 (8.5)	2 (1.7)	
**Complete Resection**		54 (96.4)	110 (94.0)	*.536*
**Grading**	*G1-G2*	7 (11.9)	34 (29.1)	*.002*
	G3	34 (57.6)	40 (34.2)	
	Gx	18 (30.5)	43 (36.8)	
**p53 ***		26 (72.2)	20 (32.8)	*<.001*
**BRCA ***		4 (20.0)	3 (7.3)	*.145*

*Intergroup comparisons between patients with adenomyosis only and patients with endometriosis were performed using the Pearson χ^2 test for categorial variables and independent t-test for continuous variables. Abbreviations: ECOG = Eastern Cooperative Oncology Group; HG = high-grade; LG = low-grade; SD = standard deviation. * relative percentages.*
